# Protected area characteristics that help waterbirds respond to climate warming

**DOI:** 10.1111/cobi.13877

**Published:** 2022-02-03

**Authors:** Elie Gaget, Alison Johnston, Diego Pavón‐Jordán, Aleksi S. Lehikoinen, Brett K. Sandercock, Alaaeldin Soultan, Luka Božič, Preben Clausen, Koen Devos, Cristi Domsa, Vitor Encarnação, Sándor Faragó, Niamh Fitzgerald, Teresa Frost, Clemence Gaudard, Lívia Gosztonyi, Fredrik Haas, Menno Hornman, Tom Langendoen, Christina Ieronymidou, Leho Luigujõe, Włodzimierz Meissner, Tibor Mikuska, Blas Molina, Zuzana Musilová, Jean‐Yves Paquet, Nicky Petkov, Danae Portolou, Jozef Ridzoň, Laimonas Sniauksta, Antra Stīpniece, Norbert Teufelbauer, Johannes Wahl, Marco Zenatello, Jon E. Brommer

**Affiliations:** ^1^ Department of Biology University of Turku Turku Finland; ^2^ International Institute for Applied Systems Analysis (IIASA) Laxenburg Austria; ^3^ Cornell Lab of Ornithology Cornell University Ithaca New York USA; ^4^ Department of Terrestrial Ecology Norwegian Institute for Nature Research (NINA) Trondheim Norway; ^5^ The Finnish Museum of Natural History University of Helsinki Helsinki Finland; ^6^ Department of Ecology Swedish University of Agricultural Sciences Uppsala Sweden; ^7^ DOPPS – BirdLife Slovenia Ljubljana Slovenia; ^8^ Department of Bioscience Aarhus University Rønde Denmark; ^9^ Research Institute for Nature and Forest Brussel Belgium; ^10^ Romanian Ornithological Society Bucharest Romania; ^11^ Instituto da Conservação da Natureza e das Florestas, IP (ICNF) Centro de Estudos de Migrações e Proteção de Aves (CEMPA) Lisbon Portugal; ^12^ Institute of Wildlife Management and Vertebrate Zoology University of Sopron Sopron Hungary; ^13^ I‐WeBS Office, BirdWatch Ireland Wicklow Ireland; ^14^ British Trust for Ornithology Thetford UK; ^15^ LPO‐BirdLife France, Fonderies Royales Rochefort France; ^16^ Department of Biology Lund University Lund Sweden; ^17^ Sovon Dutch Centre for Field Ornithology Nijmegen The Netherlands; ^18^ Wetlands International Ede The Netherlands; ^19^ BirdLife Cyprus Nicosia Cyprus; ^20^ Institute of Agricultural and Environmental Sciences Estonian University of Life Sciences Tartu Estonia; ^21^ Department of Vertebrate Ecology and Zoology, Faculty of Biology University of Gdańsk Gdańsk Poland; ^22^ Croatian Society for Bird and Nature Protection Zagreb Croatia; ^23^ Sociedad Española de Ornitología (SEO/BirdLife) Madrid Spain; ^24^ Faculty of Environmental Sciences Czech University of Life Sciences Prague Prague Czech Republic; ^25^ Département Études Aves‐Natagora Namur Belgium; ^26^ Conservation Department Bulgarian Society for the Protection of Birds Sofia Bulgaria; ^27^ Hellenic Ornithological Society Athens Greece; ^28^ SOS/BirdLife Slovakia Bratislava Slovakia; ^29^ Lithuanian Ornithological Society Vilnius Lithuania; ^30^ Institute of Biology University of Latvia Salaspils Latvia; ^31^ BirdLife Österreich Vienna Austria; ^32^ Dachverband Deutscher Avifaunisten e.V. (DDA) Federation of German Avifaunists Münster Germany; ^33^ Istituto Superiore per la Protezione e la Ricerca Ambientale (ISPRA) Ozzano dell'Emilia Italy

**Keywords:** climate adaptation, colonization, conservation policy, distribution change, EU Birds Directive, LIFE program, wetland, adaptación climática, cambios en la distribución, colonización, Directiva de Aves de la UE, humedal, políticas de conservación, programa LIFE

## Abstract

Protected area networks help species respond to climate warming. However, the contribution of a site's environmental and conservation‐relevant characteristics to these responses is not well understood. We investigated how composition of nonbreeding waterbird communities (97 species) in the European Union Natura 2000 (N2K) network (3018 sites) changed in response to increases in temperature over 25 years in 26 European countries. We measured community reshuffling based on abundance time series collected under the International Waterbird Census relative to N2K sites’ conservation targets, funding, designation period, and management plan status. Waterbird community composition in sites explicitly designated to protect them and with management plans changed more quickly in response to climate warming than in other N2K sites. Temporal community changes were not affected by the designation period despite greater exposure to temperature increase inside late‐designated N2K sites. Sites funded under the LIFE program had lower climate‐driven community changes than sites that did not received LIFE funding. Our findings imply that efficient conservation policy that helps waterbird communities respond to climate warming is associated with sites specifically managed for waterbirds.

## Abstract 2

*Article impact statement*: Protected area management and species targets help waterbird communities respond to climate warming inside the Natura 2000 network.

## Resumen

Las redes de áreas protegidas ayudan a las especies a responder al calentamiento climático. Sin embargo, se sabe muy poco sobre la contribución de las características ambientales y relevantes para la conservación de un sitio a estas respuestas. Investigamos cómo la composición de las comunidades no reproductivas de aves acuáticas (97 especies) en la red (3,018 sitios) Natura 2000 de la Unión Europea (N2K) cambió en respuesta a los incrementos de la temperatura durante más de 25 años en 26 países europeos. Medimos la reorganización comunitaria con base en series temporales de abundancia recolectadas durante el Censo Internacional de Aves Acuáticas en relación con los objetivos de conservación de los sitios N2K, el periodo de asignación de fondos y el estado del plan de manejo. La composición comunitaria de las aves acuáticas en los sitios con planes de manejo y designados explícitamente para su protección cambió más rápidamente en respuesta al calentamiento climático que en otros sitios N2K. Los cambios comunitarios temporales no se vieron afectados por el periodo de asignación a pesar de una mayor exposición al incremento de la temperatura dentro de los sitios N2K de asignación tardía. Los sitios financiados por el programa LIFE tuvieron menos cambios comunitarios causados por el clima que los sitios que no recibieron este financiamiento. Nuestros hallazgos sugieren que la política de conservación eficiente que ayuda a las comunidades de aves acuáticas a responder al calentamiento climático está asociada con sitios específicamente gestionados para las aves acuáticas.

## INTRODUCTION

Conservation policies have historically aimed to stop or mitigate species extinction, habitat degradation, and natural resource depletion. A major new conservation objective is to facilitate species responses to climate warming (Rannow et al., 2014; van Teeffelen et al., 2015). This includes enabling species movement through a landscape so that species can track their climatic niches to prevent populations being unable to shift their distributions away from places that no longer have a suitable climate. The need for this conservation objective has become pressing as species’ distribution changes lag behind the velocity of climate warming (Devictor et al., 2012; Lenoir et al., 2020), increasing the risk of mismatch between species’ past climatic niches and current abiotic conditions (Essl et al., 2015). Time lags in organismal responses are exacerbated by anthropogenic pressures, mainly due to habitat degradation (Auffret & Thomas, 2019; Gaget et al., 2020; Schinegger et al., 2016) and overexploitation (Engelhard et al., 2014; Lenoir et al., 2020).

Protected area (PA) networks can facilitate community changes in response to climate warming (Gaüzère et al., 2016; Lehikoinen et al., 2019; Thomas et al., 2012). Species extending their distribution toward cold margins more often colonize PAs (Thomas et al., 2012), resulting in changes to the overall community composition in PAs according to species’ thermic affinities (Gaget et al., 2021). However, these effects are not consistent across PAs (Gaget et al., 2021), and it is unclear whether PA characteristics contribute to a PA being effective in facilitating such responses to climate warming (van Kerkhoff et al., 2019, but see Lawson et al., 2014; Wessely et al., 2017). Individual PAs differ in when and why they were established, management planning, and funding base, among other factors. These sites characteristics are important to achieving conservation targets (Rodrigues & Cazalis, 2020) and might also facilitate species responses to increasing temperature (Lawson et al., 2014; Wessely et al., 2017). Although management, and financial resources allocated to management, may facilitate species distribution shifts by reducing anthropogenic pressures, species‐specific management may also increase species persistence by maintaining preexisting habitat conditions despite climate warming or by mitigating the negative impacts of the temperature changes (Greenwood et al., 2016). Therefore, PAs facilitate species distribution changes, but may also reduce local extirpation (Gaget et al., 2021; Peach et al., 2019). Pinpointing which conservation policies help species respond to climate warming in both ways can help refine climate resilient PA networks.

We examined the European Union's (EU) Natura 2000 (N2K) network to investigate whether climate‐driven community changes were positively influenced by early designation, having a management plan, targeting a focal community, or having specific funding. The N2K network was established specifically for biodiversity conservation and is the backbone of the EU strategic aim to maintain and restore European biodiversity according to the Birds and Habitats Directives (2009/147/EC and 92/43/EEC, respectively). The Birds Directive is designed to protect wild populations of birds in the EU, whereas the Habitats Directive focuses on the conservation of other species and their habitats. The effectiveness of the N2K network for mitigating the negative effects of climate warming is thought to be limited by insufficient and misallocated funding from the EU's LIFE program (L'Instrument Financier pour l'Environnement), which is dedicated to N2K network conservation (Hermoso et al., 2017; Lung et al., 2014), and a lack of site management (Hochkirch et al., 2013). Therefore, it is important to evaluate how a management plan and funds are associated with the ability of PAs to facilitate climate‐driven community changes.

We evaluated the community reshuffling of nonbreeding waterbird communities at sites inside the N2K network over 25 years. Distribution changes of nonbreeding waterbirds are highly dynamic (Lehikoinen et al., 2013; Maclean et al., 2008; Pavón‐Jordán et al., 2019), and PAs are important for waterbird conservation (Amano et al., 2018) and response to climate warming (Gaget et al., 2021; Pavón‐Jordán et al., 2015). We investigated how waterbird communities changed over time in response to climate warming, relative to 4 N2K site characteristics. We expected the studied characteristics to affect how a waterbird's community temperature index (CTI) varied over time in response to temperature increase. Our main hypothesis was that having 1 or more of the characteristics results in a more positive changes in CTI in response to temperature changes and to a positive CTI standard deviation (CTI_SD_) trend, due to a disproportional increase in warm‐dwelling species (Gaget et al., 2021). Our alternative hypothesis was that having a management plan or LIFE funding slows the temporal change in CTI because of a persistence of cold‐dwelling species enticed to remain at a site by dedicated conservation actions.

## METHODS

**FIGURE 1:**
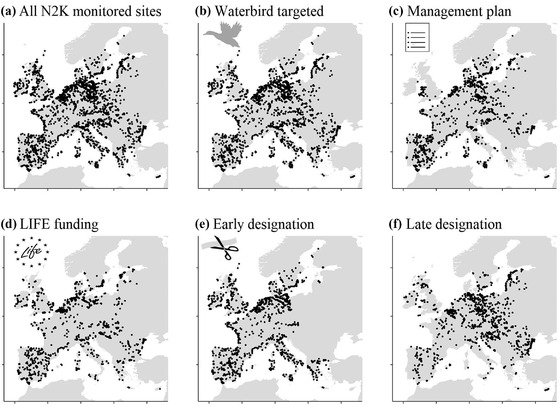
Survey sites (a) in a protected area (PA) under the Natura 2000 (N2K) network scheme included in this study (*n* = 3018). The N2K characteristics include (b) waterbirds targeted, (c) management plan prepared, (d) LIFE funding obtained, and site designation is (e) early or (f) late (early before 2000, the middle year based on PA designation period or 1982–2017)

We used abundance data for 97 nonbreeding waterbird species gathered at 3018 N2K sites from 1993 to 2017 from the International Waterbird Census (IWC) (Delany, 2010) conducted in 26 EU Member States (Figure 1a) (including the United Kingdom) (Appendix S1). The IWC, coordinated by Wetlands International (www.wetlands.org), is conducted once a year in January by skilled ornithologists following a standardized survey protocol. An IWC site was considered to represent an N2K site if the central IWC coordinates fell within the polygon of a N2K site (www.eea.europa.eu). This approach resulted in a reasonable overlap, with on average (SD) 80.8% (23.3) of the IWC surface included in the corresponding N2K site (based on validation with 1307 IWC sites with available polygons). Not all IWC sites were surveyed every year, and because our aim was to quantify temporal community changes, we considered only sites with ≥5 surveys and ≥2 species per survey. All species included in the analyses overwinter in the Western‐Palearctic and are listed as targeted by N2K designation, referred to in Article 4 of the Birds Directive (Appendix S2). The abovementioned criteria resulted in a data set of 38,559 surveys of 3018 sites. The cumulative record was 199 million birds from 97 species over 25 years.

The N2K site characteristics were collated from the N2K and the LIFE program databases (Appendix S2). These document whether waterbirds were targeted (yes or no), a management plan was prepared (yes or no), and LIFE funding was obtained (yes or no) and the period during which protection was first designated (early or late) (early before 2000, the mid‐year according to PA designation period or 1982–2017) (Figure 1 & Appendix S2). We treated all these characteristics as binary (e.g., yes or no). Because all the studied species are targeted by the Birds Directive but not by the Habitats Directive, we considered that a PA targets waterbirds if the designation was under the Birds Directive. The existence of a site management plan (the only information about management in the N2K database) is necessary for its implementation, but does not confirm that management was conducted. We thereby assumed that any association we found between a management plan and our response would be an underestimated effect of the actual site management. We focused on the LIFE program for funding because it is the most dedicated to N2K conservation, but the N2K network is supported by other agencies including the European Agricultural Fund for Rural Development and Structural and Cohesion Funds. For each site, annual winter temperatures were computed over the nonbreeding period, which likely influences waterbird location in January, as the average of the mean monthly temperatures of November, December, and January in the HadCRUT4 data set (Morice et al. 2012, spatial resolution of 0.5°, www.cru.uea.ac.uk).

We measured the community response to climate warming by calculating the CTI (Devictor et al., 2008) and its SD (Gaget et al., 2021), based on species abundance. We used nonbreeding waterbird species temperature indices (STI) from Gaget et al. (2021). The STI of a given species corresponded to the long‐term average temperature in January (1950–2000, www.worldclim.org, spatial resolution of 0.25°) calculated across its nonbreeding distribution (BirdLife International & HBW, 2017). The CTI of 1 site in a given survey corresponded to the mean STI of all species present in that site in that survey weighted by their log_e_(abundance + 1) to reduce the influence of the highly abundant waterbird species (Godet et al., 2011). A CTI increase can be caused by an increasing abundance of species with high STIs or by a decreasing abundance of species with low STIs. The CTI_SD_ represented the standard deviation around the CTI, assessed from the species STI present in the community and weighted by the log_e_(abundance + 1). When the CTI trend is positive, a positive CTI_SD_ trend suggests that increases in warm‐dwelling species exceed the relative decreases of cold‐dwelling species. A positive CTI trend with a negative CTI_SD_ trend suggests that decreases in cold‐dwelling species exceed the relative increases of warm‐dwelling species (Gaget et al., 2021).

### Statistical analyses

We evaluated how waterbird thermal communities changed over time according to N2K characteristics by assessing the temporal trends of CTI, CTI_SD_, and temperature in relation to the N2K characteristics. We used linear mixed‐effects models with CTI, CTI_SD_, or temperature as the response. Fixed effects were the N2K site characteristics (waterbirds target, management plan, LIFE funding, and designation period) and 2‐way interactions between year and each of the N2K characteristics. The site and the country were added as random effects, and the spatial autocorrelation was taken into account by including an exponential spatial correlation structure in the model (Gaget et al., 2018). Then, the temporal trends of CTI, CTI_SD_, and temperature were estimated separately for each of the 16 possible combinations of characteristics and compared with each other in a post hoc analysis with a Bonferroni correction.

We measured the climatic debt accumulated by waterbird communities for each N2K characteristics as the difference between temperature increase and CTI increase (Devictor et al., 2008). We assessed both temperature and CTI spatial gradients by measuring their latitudinal gradient with a linear model and then converting them into kilometer gradients (divided by 111.128 [i.e., the average kilometers per 1 decimal degree latitude over the study area]). Then, we assessed the velocity of both temperature and CTI changes (kilometers per year) from their temporal trends (degrees Celsius per year) and their spatial gradients (degrees Celsius per kilometer). The spatial climatic debt was the difference between CTI velocity and temperature velocity (both kilometers per year).

We conducted 4 sensitivity analyses (Appendix S3) to evaluate the robustness of our results to a number of analytical decisions. We checked whether CTI and CTI_SD_ trends were overly influenced by a few abundant species by using species occurrence instead of abundance; whether the CTI and CTI_SD_ trends were affected by the geographical west‐east EU accession gradient by fitting models only with the subset of 11 countries in the EU before 1992 (*n* = 2186 sites); whether the community changes resulted from a decrease or an increase of species richness; and whether the CTI trends associated with each N2K site characteristic were correlated with the amount of protected wetland surface.

All statistical analyses were performed with R.3.6.2 (R Core Team, 2019) with the glmmTMB package (Magnusson et al., 2017). We used *emmeans* to assess the CTI temporal trend for N2K characteristics and the post hoc tests (Lenth et al., 2018).

## RESULTS

There was considerable variation in the characteristics across the N2K sites (Figure 1). Almost 82% of sites were designated specifically for waterbirds (Figure 1b), 43% had a management plan (Figure 1c), 50% received LIFE funding (Figure 1d), and 46% were designated after 2000 (Figure 1f). The proportion of PAs with a management plan was almost identical among PAs with (44%) and without (39%) waterbird targets and between PAs early (46%) and late (40%) designation.

**FIGURE 2:**
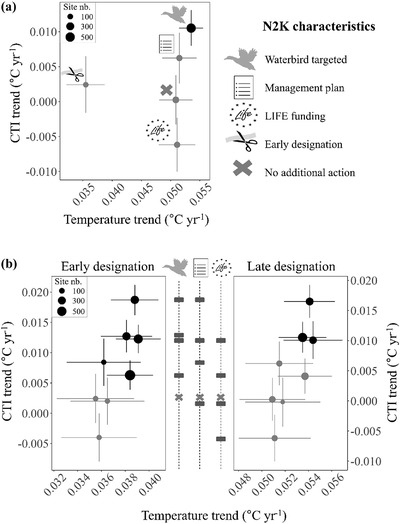
Estimated temporal trends of community temperature index (CTI) and temperature in survey sites protected under the Natura 2000 (N2K) network (black, CTI trends that differ significantly from 0; gray, CTI trends that do not differ significantly from 0; lines, SE; dot size, proportional to number of sites [reported in Table 1]): (a) main effects of site characteristics (i.e., waterbirds targeted, management plan, LIFE funding, early designation [before 2000], and none of these characteristics) and (b) temporal trends in CTI and temperature for all the possible combinations of N2K characteristics (bars between graphs, trends separated by characteristic; point showing fastest temporal trend, sites targeting waterbirds with a management plan; second point from the top, sites targeting waterbirds, etc.). To facilitate visual interpretation, *y*‐axes differ between sites designated early and late

Temperatures increased on average across all categories of the IWC sites in the N2K network, and all increases were statistically significant (Figure 2). There was a markedly slower increase in temperature at N2K sites established before 2000 compared with those established after 2000 (*t* = 7.1, *p* < 0.001), consistent with the geographical west‐east EU accession gradient (Figure 1f & Appendix S3) and European west‐east gradient of temperature increase over the same period (Gaget et al., 2021). However, the temperature increase was similar across all the other site characteristics (Figure 2a). In contrast to temperature changes, the community adjustment as quantified by the CTI temporal trend differed substantially across N2K site characteristics (Table 1). The trend for increasing CTI through time was only significant in N2K sites targeted to protect waterbirds (Figure 2a), but not in PAs with only a management plan, an early designation, EU LIFE funding, or the absence of all of these characteristics (Figure 2a). Looking at the combinations of characteristics (Figure 2b, Table 1, & Appendix S4), we found that if a site characteristic was not combined with another, PAs designated for waterbirds had the strongest community adjustment (significant in sites with early and late designation) (Figure 2b), followed by PAs with a management plan (significant in sites with early but not late designation) (Figure 2b). In contrast, LIFE funding alone was not associated with climate‐driven community adjustment in PAs (Figure 2b). Furthermore, PAs designated for waterbirds and that had management plans were associated with greater community adjustment (Figure 2b). Surprisingly, combinations of site characteristics, including LIFE funding, were not associated with community adjustment to climate warming (Figure 2b). The CTI_SD_ trends were only strictly positive at early designated sites, whatever the combination of other characteristics (Table 1). Consequently, when the CTI increased in early designated PAs, the increase in warm‐dwelling species abundance exceeded the decrease of cold‐dwelling species (Figure 2b, left). In late‐designated PAs, when the CTI increased, community changes were likely related to both increase in warm‐dwelling species and decrease in cold‐dwelling species (Figure 2b, right). Otherwise, in the absence of significant CTI and CTI_SD_ trends, if the community composition changed over time it was not related on average to species thermal affinities.

**TABLE 1:** Temporal trends in the community temperature index (CTI) and the CTI_SD_ relative to climatic debt, the number of survey sites (*n*), and Natura 2000 (N2K) site characteristics and time of designation (early, before 2000; late, after 2000)

	Trend in °C/year (95% CI)			Debt (km)		*n*	
N2K characteristic	Index	Early (<2000)	Late (>2000)	Early	Late	Early	Late
–	CTI	0.019 (0.014 to 0.024)^a^	0.000 (−0.007 to 0.007)	223	351	55	134
	CTI_SD_	0.014 ((0.007 to 0.022)^a^	0.005 [−0.002 to 0.011)				
W	CTI	0.002 (−0.005 to 0.010)	0.011 (0.006 to 0.016)^a^	140	268	263	395
	CTI_SD_	0.014 (0.009 to 0.019)^a^	0.004 (−0.001 to 0.009)				
MP	CTI	0.013 (0.007 to 0.018)^a^	0.006 (−0.001 to 0.013)	169	297	47	95
	CTI_SD_	0.014 (0.006 to 0.021)^a^	0.004 (−0.003 to 0.011)				
LIFE	CTI	0.008 (0.001 to 0.016)^a^	–0.006 (−0.014 to 0.001)	288	415	49	96
	CTI_SD_	0.015 (0.008 to 0.022)^a^	0.005 (−0.002 to 0.012)				
W + MP	CTI	–0.004 (−0.012 to 0.004)	0.017 (0.011 to 0.022)^a^	86	214	286	226
	CTI_SD_	0.013 (0.009 to 0.018)^a^	0.004 (−0.002 to 0.008)				
W + LIFE	CTI	0.006 (0.002 to 0.011)^a^	0.004 (−0.002 to 0.010)	205	333	524	197
	CTI_SD_	0.014 (0.010 to 0.019)^a^	0.005 (−0.001 to 0.01)				
MP + LIFE	CTI	0.002 (−0.006 to 0.010)	0.000 (−0.008 to 0.008)	234	362	50	22
	CTI_SD_	0.014 (0.007 to 0.021)^a^	0.004 (−0.003 to 0.012)				
W + MP + LIFE	CTI	0.012 (0.008 to 0.017)^a^	0.010 (0.004 to 0.016)^a^	151	279	369	210
	CTI_SD_	0.014 (0.009 to 0.018)^a^	0.004 (−0.002 to 0.01)				

Abbreviations: LIFE, LIFE program funding obtained; MP, management plan prepared; W, waterbirds targeted.

^a^ Significantly different from 0. Pairwise comparisons are in Appendix S4.

The temperature latitudinal gradient was about −0.36°C per 100 km (−0.40°C per latitudinal degree; SE 0.01, *t* = −51.89, *p* < 0.001) and the latitudinal gradient for CTI was about −0.26°C per 100 km (−0.28°C per latitudinal degree; SE 0.01, *t* = −31.41, *p* < 0.001). Thus, on average a northward shift of 100 km was equivalent to a reduction in average temperature of −0.36°C and an average CTI of −0.26°C. Converting the temporal trends to spatial velocity revealed an overall climatic debt of over 257 km in 25 years across all sites. According to the faster temperature increase in late‐designated sites, the climatic debt was twice as high in late compared with early designated sites (Table 1). The expected average climatic debt varied from 86 km in early designated sites targeted to protect waterbirds with a management plan to 415 km in late‐designated sites with only LIFE funding (Table 1).

Our sensitivity analyses (Appendix S3) showed that the CTI and CTI_SD_ trends were fairly consistent if based on occurrence instead of abundance; the CTI and CTI_SD_ trends remained mostly unchanged considering all the EU countries or just the subset of countries that joined the EU before 1992; the species richness increase was significant for each combination of N2K characteristics and trends of species richness were correlated with CTI trends; and CTI increases were positively correlated with protected wetland surface area, but including the surface as a covariate did not qualitatively change the results described above.

## DISCUSSION

We found that N2K sites designated for waterbirds were characterized by faster responses of the waterbird communities to increasing winter temperature. The response was particularly strong in N2K sites targeting waterbirds that also had a management plan. However, despite the clear climate‐driven community adjustment, the temperature increase was 2–4 times faster than the community waterbird response, resulting in a large climatic debt. Such lags are common for terrestrial taxa (Lenoir et al., 2020) and are typically viewed as an insufficient distribution change in response to climate warming (Devictor et al., 2012). Our findings suggest that the most efficient, although perhaps not sufficient, conservation policy to help waterbird communities adjust to climate warming is to protect sites that are suitable for waterbirds and develop a plan for managing the protected sites.

Protection of sites under the N2K scheme is typically based on recognizing that certain sites are of ecological importance for conservation of particular species or habitats. Our findings demonstrate that sites designated for waterbirds indicate a capacity for the sites to maintain—and enhance—the species richness of these waterbird communities (Appendix S3) and to accommodate a more dynamic climate‐driven community change. Indeed, sites in which the CTI increased rapidly were also sites where the number of species increased rapidly, demonstrating a directionality wherein community changes were driven by colonization of warm‐dwelling species and likely not by extinction of cold‐dwelling species. A colonization‐driven community change in protected landscapes appears to be common in birds (Gaget et al., 2021; Lehikoinen et al., 2019) and invertebrates (Thomas et al., 2012).

Slightly unexpectedly, we found a negative relationship between LIFE funding and community response to climate warming. This supports the alternative hypothesis, in which LIFE funding is specifically aimed at improving the persistence of a focal species (more than in the general management plan), but those species‐specific conservation measures do not provide benefits for other warm‐dwelling species extending their distribution north into the site. Alternatively, sites receiving LIFE funding may have major threats, presenting a critical conservation issue (Lung et al., 2014) in which community adjustment to climate warming is limited because of habitat degradation (Auffret & Thomas, 2019; Gaget et al., 2020; Schinegger et al., 2016). Hence, it may be that substantial conservation funding has been allocated to PAs that are degraded or threatened (but see Hermoso et al. [2017]). For example, from 1992 to 2016, LIFE funding allocated to reed‐bed conservation (i.e., restoration or prevention of degradation from drainage, pollution, and destruction) was about €56 million (Giakoumi et al., 2019). We did not collect information on which species or which sites were targeted by LIFE‐funded projects. Further investigation would be welcome to establish cost‐effective assessments of conservation measures regarding species‐specific adaptation to climate warming.

Our findings showed that the main tool to enforce conservation measures, the management plan, was associated with faster community adjustment to climate warming in early designated sites. The presence of a management plan indicates potential active involvement of site managers. Having a management plan for N2K PAs is not mandatory in every EU Member State, but it is strongly encouraged by Articles 4 and 6 of the Birds and Habitats Directives, respectively. Establishing a management plan is an important step to identify the environmental and socioeconomic issues with the stakeholders and elaborate conservation measures to maintain the targeted species or habitats to a favorable conservation status. A clear and intuitive finding was that the community adjustment was faster in sites with a management plan and that targeted a focal community. The implementation of management measures may support the ecological processes of climate‐driven distribution change (Lawson et al., 2014) by reducing the additional negative impacts of land‐use change (Wessely et al., 2017) or disturbance (Väänänen, 2001). Adequate wetland management dedicated to waterbirds may provide suitable conditions for species extending their distribution (Ausden, 2014). Such effect has been empirically assessed for butterflies (Lawson et al., 2014), where habitat management promoting a focal community (i.e., livestock grazing for threatened butterfly species) facilitates range expansion of a butterfly not directly targeted by the conservation measures. Apart from this “facilitation” process, species‐specific management may improve species persistence (Greenwood et al., 2016). For instance, Pearce‐Higgins et al (2011) demonstrate that adapted habitat management ensures the local persistence of the golden plover (*Pluvialis apricaria*) despite its vulnerability to temperature increase. In our case, for example, a management plan could facilitate the persistence of some cold‐dwelling species, which then translates to an increase in the mean climatic debt of the community.

Importantly, however, we lacked information on whether and to what extent a management plan has actually been implemented because publicly available records only denote whether a management plan has been prepared. It therefore remains possible that N2K sites for which a management plan exists (42% in this study) represent a subset that—for whatever reason—shows faster community responses to climate warming. To ascertain whether it is indeed the (costly) management on the ground that benefits community adjustment to climate warming (Lawson et al., 2014), future work should directly contrast sites in which management has been implemented with those where a plan merely exists. To allow critical evaluation of this conservation policy, further development of N2K reporting should include information on implementation of management plans (e.g., Pearce‐Higgins et al., 2011).

Interestingly, we found no evidence of an association between the designation period and CTI change. Our expectation was that early PA designation would improve site conservation according to the Habitats and Birds Directives targets (i.e., to maintain or restore habitats and species population at a favorable conservation status), with subsequent positive effects of site conservation on CTI trends (Gaüzère et al., 2016). This result suggests that PA designation itself (i.e., the designation time) is not associated with more positive CTI changes in response to climate warming. Indeed, depending on political and stakeholder supports, N2K designation alone does not suffice to achieve conservation goals (Kati et al., 2015). However, our findings regarding the CTI_SD_ trends suggest that early PA designation is positively associated with cold‐dwelling species persistence. Nevertheless, species‐specific models would be more suitable to investigate this pattern. Overall, despite the absence of immediate effects of the designation period on the CTI trend, early designation might still benefit species vulnerable to climate warming.

The importance of the EU Birds Directive to facilitate waterbird responses to climate warming is perceptible in EU and non‐EU countries (Gaget et al., 2018; Pavón‐Jordán et al., 2020), as well as inside and outside the N2K network (Pavón‐Jordán et al., 2015). We demonstrated that community adjustment to climate warming was heterogeneous within the N2K network, but that protection of sites targeting waterbirds may help their communities adjust to climate warming and that this adjustment was faster with a management plan. However, our results are correlative; determining causal mechanisms would require further species‐specific investigations to directly assess effects of these site characteristics on species demographic parameters. Also, waterbird communities might be influenced by other environmental changes, possibly resulting in complex interacting effects with conservation efforts. For example, changes in precipitation and conservation measures can affect water levels, which in turn can affect waterbird feeding strategies and community compositions (Holm & Clausen, 2006).

The N2K network presents a great opportunity to shift goals toward a climate‐resilient network. Historically, PA designation has mostly focused on maintaining and improving local biodiversity targeted by the Birds and Habitats Directives. The observed waterbird climatic debt, averaging 250 km over 25 years, highlights a lag of biological responses to climate warming and the need for dynamic conservation targets. Habitat connectivity is a major goal to facilitate species dispersal despite human‐caused barriers (Lawler et al., 2013). Habitat connectivity is already targeted in the EU Biodiversity Strategy for 2030; it is one of the central parameters for building a truly coherent Trans‐European Nature Network (TEN‐N) based on the N2K network (European Commission, 2020). However, considering that waterbird dispersal capacities are largely unlimited in Europe (e.g., Gourlay‐Larour et al., 2012), our results suggest that PA management should be carefully considered to achieve a climate‐resilient network.

With that aim, more empirical evidence is required to explore the effectiveness of conservation measures and to inform the ambitious EU 2030 Biodiversity Strategy. Despite the outstanding intergovernmental organization that provides structure for the N2K network, the lack of standardized reports on N2K site conservation measures jeopardizes progress in quantifying conservation outcomes. Basic and critical information on conservation measures, threats, targets, budgeting, and the spatial and temporal extent of conservation measures is required for a more detailed assessment (Rodrigues & Cazalis, 2020). The Biodiversity Strategy for 2030 emphasizes the need to help species to adapt to climate warming, which is welcome in view of the expected negative impacts of climate warming on waterbirds (Nagy et al., 2021). We suggest that a first move is binding establishment of on‐site management planning by EU Member States, including follow‐up on management plan implementation, to facilitate species communities adjusting to climate warming.

## Supporting information

Appendix 1. Species information
Table S1. List of the species with their species temperature index (STI) and total number of birds counted over the 25‐year period.
Appendix 2. Natura 2000 (N2K) network site characteristics.
Appendix 3. Sensitivity analyses.
Figure S1: Parameter estimates (±95%CI) of the temporal trends of a) CTI, b) CTI_SD_ and c) temperature between N2K characteristics, based on all countries (black) or only the EU Member States before 1992 (grey), using abundance (filled dots) or occurrence data (unfilled dots)
Figure S2: Temporal trend (±CI95%) of CTI (in grey) and species richness (R, in black) per combination of N2K protective action
Figure S3. Temporal trend (±CI95%) of CTI and species richness per combination of N2K protective action
Appendix 4. Pairwise comparisons of the CTI temporal trends according to the N2K characteristics; waterbird(s) were targeted (W), a management plan has been prepared (MP), the period the protection was designated (Late or Early, where early is <2000), or LIFE funding has been obtained (LIFE). The significant differences, after Bonferroni correction, are denoted in bold (α<0.05)
