# Supplementary material for: Protected area characteristics that help waterbirds respond to climate warming
Source: Conserv Biol. 2022 Feb 3;36(4):e13877. doi: 10.1111/cobi.13877 (PMC10286641; doi:10.1111/cobi.13877)
Supplement: Supplementary file 1 — Appendix 1. Species information Table S1. List of the species with their species temperature index (STI) and total number of birds counted over the 25‐year period. Appendix 2. Natura 2000 (N2K) network site characteristics. Appendix 3. Sensitivity analyses. Figure S1: Parameter estimates (±95%CI) of the temporal trends of a) CTI, b) CTISD and c) temperature between N2K characteristics, based on all countries (black) or only the EU Member States before 1992 (grey), using abundance (filled dots) or occurrence data (unfilled dots) Figure S2: Temporal trend (±CI95%) of CTI (in grey) and species richness (R, in black) per combination of N2K protective action Figure S3. Temporal trend (±CI95%) of CTI and species richness per combination of N2K protective action Appendix 4. Pairwise comparisons of the CTI temporal trends according to the N2K characteristics; waterbird(s) were targeted (W), a management plan has been prepared (MP), the period the protection was designated (Late or Early, where early is <2000), or LIFE funding has been obtained (LIFE). The significant differences, after Bonferroni correction, are denoted in bold (α<0.05) [file COBI-36-0-s001.docx]

Pinpointing which protected area characteristics help community response to climate warming: waterbirds in the European Union’s Natura 2000 network

**Appendix 1. Species information**

The International Waterbird Census (IWC) targets all waterbird species since the end of the 1980s. Long-term monitoring has been conducted in Austria, Belgium, Bulgaria, Croatia, Cyprus, Czechia, Denmark, Estonia, Finland, France, Germany, Greece, Hungary, Ireland, Italy, Latvia, Lithuania, Netherlands, Poland, Portugal, Romania, Slovakia, Slovenia, Spain, Sweden and United Kingdom. However, gulls and shags were not systematically included in some national monitoring. A complete census was performed later in Romania (1999), Belgium (Flandre, 2000), Denmark (2001), United Kingdom (2002), Ireland (2002) and Sweden (still not full). We corrected the CTI to avoid a bias induced by the addition of these species over time, by centring CTI values per site (not reducing) before the monitoring change and adding these values to the average site CTI value of the years after the monitoring change (Gaget et al. 2020b). The CTI_SD_ was not corrected (but see Gaget et al. 2020b).

Table S1. List of the species with their species temperature index (STI) and total number of birds counted over the 25-year period.

| Species name | STI | Total abundance | Species name | STI | Total abundance |
| --- | --- | --- | --- | --- | --- |
| *Actitis hypoleucos* | 23.157 | 13882 | *Ichthyaetus melanocephalus* | 9.306 | 216165 |
| *Anas acuta* | 16.897 | 1598034 | *Larus argentatus* | 4.578 | 6695899 |
| *Anas crecca* | 12.725 | 8708854 | *Larus canus* | 1.618 | 1713090 |
| *Anas platyrhynchos* | -0.020 | 20788467 | *Larus fuscus* | 18.580 | 999113 |
| *Anser albifrons* | 2.533 | 8841023 | *Larus marinus* | -2.492 | 261923 |
| *Anser anser* | 4.465 | 5105551 | *Limosa lapponica* | 19.227 | 916125 |
| *Anser brachyrhynchus* | 2.021 | 1111122 | *Limosa limosa* | 21.015 | 1368458 |
| *Anser erythropus* | 2.434 | 1355 | *Mareca penelope* | 16.530 | 15085930 |
| *Anser fabalis & serrirostris* | -2.514 | 3024771 | *Mareca strepera* | 11.735 | 1228652 |
| *Ardea cinerea* | 18.734 | 489794 | *Marmaronetta angustirostris* | 5.954 | 296 |
| *Arenaria interpres* | 17.861 | 411282 | *Melanitta fusca* | 0.670 | 389784 |
| *Aythya ferina* | 11.336 | 4660585 | *Melanitta nigra* | 2.280 | 1756485 |
| *Aythya fuligula* | 10.452 | 6720177 | *Mergellus albellus* | -1.579 | 215464 |
| *Aythya marila* | 0.428 | 2105733 | *Mergus merganser* | -0.397 | 787884 |
| *Aythya nyroca* | 9.773 | 16767 | *Mergus serrator* | -1.083 | 381289 |
| *Botaurus stellaris* | 18.395 | 2927 | *Microcarbo pygmeus* | 2.744 | 264738 |
| *Branta bernicla* | 2.863 | 6788806 | *Netta rufina* | 5.352 | 418354 |
| *Branta leucopsis* | 1.686 | 3390313 | *Numenius arquata* | 18.888 | 2959783 |
| *Branta ruficollis* | 1.097 | 417362 | *Numenius phaeopus* | 22.058 | 11416 |
| *Bubulcus ibis* | 22.960 | 223166 | *Nycticorax nycticorax* | 23.472 | 13259 |
| *Bucephala clangula* | -1.105 | 1766062 | *Oxyura leucocephala* | 1.270 | 11751 |
| *Calidris alba* | 18.862 | 496630 | *Pelecanus crispus* | 8.366 | 35467 |
| *Calidris alpina* | 11.831 | 13648117 | *Pelecanus onocrotalus* | 22.005 | 353 |
| *Calidris canutus* | 19.078 | 3839071 | *Phalacrocorax aristotelis* | 2.299 | 31441 |
| *Calidris maritima* | -2.483 | 19034 | *Phalacrocorax carbo* | 18.408 | 2925140 |
| *Calidris minuta* | 22.774 | 148258 | *Philomachus pugnax* | 22.963 | 33438 |
| *Casmerodius albus* | 21.856 | 186057 | *Phoenicopterus roseus* | 20.584 | 1648943 |
| *Charadrius alexandrinus* | 19.198 | 131230 | *Platalea leucorodia* | 17.950 | 41599 |
| *Charadrius hiaticula* | 22.108 | 383072 | *Plegadis falcinellus* | 23.137 | 50634 |
| *Chroicocephalus genei* | 11.898 | 67716 | *Pluvialis apricaria* | 5.357 | 2459930 |
| *Chroicocephalus ridibundus* | 6.582 | 9416624 | *Pluvialis squatarola* | 18.836 | 1421314 |
| *Ciconia ciconia* | 22.949 | 41713 | *Podiceps auritus* | 3.394 | 11078 |
| *Clangula hyemalis* | -2.500 | 1119744 | *Podiceps cristatus* | 10.083 | 1598172 |
| *Cygnus columbianus* | 2.437 | 119929 | *Podiceps grisegena* | 2.273 | 2646 |
| *Cygnus cygnus* | -1.491 | 456924 | *Podiceps nigricollis* | 17.998 | 448628 |
| *Cygnus olor* | 1.269 | 1551078 | *Porphyrio porphyrio* | 22.120 | 30385 |
| *Egretta garzetta* | 21.263 | 358354 | *Rallus aquaticus* | 4.524 | 25572 |
| *Fulica atra* | 5.865 | 16106076 | *Recurvirostra avosetta* | 22.045 | 918636 |
| *Fulica cristata* | 22.590 | 1054 | *Somateria mollissima* | -7.529 | 3838707 |
| *Gallinago gallinago* | 18.584 | 144440 | *Spatula clypeata* | 14.912 | 2612133 |
| *Gallinula chloropus* | 16.307 | 359493 | *Sterna sandvicensis* | 12.381 | 28107 |
| *Gavia arctica* | 0.764 | 14561 | *Tachybaptus ruficollis* | 18.596 | 391187 |
| *Gavia stellata* | 3.054 | 27516 | *Tadorna ferruginea* | 8.880 | 5595 |
| *Grus grus* | 14.716 | 1206396 | *Tadorna tadorna* | 4.300 | 3048067 |
| *Haematopus ostralegus* | 15.341 | 6991052 | *Tringa erythropus* | 21.035 | 29441 |
| *Himantopus himantopus* | 22.126 | 88522 | *Tringa nebularia* | 23.157 | 30159 |
| *Hydrocoloeus minutus* | 3.700 | 9639 | *Tringa ochropus* | 21.099 | 11092 |
| *Ichthyaetus audouinii* | 11.453 | 15478 | *Tringa totanus* | 15.484 | 1011945 |
|  |  |  | *Vanellus vanellus* | 4.517 | 7245956 |

**Appendix 2. Natura 2000 (N2K) network site characteristics.**

Site characteristics were collected from eunis.eea.europa.eu/sites and ec.europa.eu/easme/en/life.

Waterbird targeted (n_Yes_=2470, n_No_=548 IWC sites). The N2K site designation may target bird species listed in Annex I of the Birds Directive and migratory species not listed in Annex I (Birds Directive, Article 4). All waterbird species studied are migratory species apart from the Red-knobbed Coot (*Fulica cristata*) and the Purple Swamphen (*Porphyrio porphyrio*) (Birdlife 2019), which are however listed in Annex I of the Birds Directive. A N2K site can be established without a waterbird target when the designation is under the Habitats Directive.

Management plan (n_Yes_=1305, n_No_=1713 IWC sites). The management plan of a N2K site is made by the relevant stakeholders to define the objectives needed to maintain or restore the conservation status of the habitats and species of community interest. Management plan date and measures (like habitat restoration, eradication of invasive species, translocation, prescribed burning or water management) are lacking from the site information in the large majority of the sites. We considered the management plan “In preparation” as not existing.

LIFE funding (n_Yes_=1517, n_No_=1501 IWC sites). From 1992 to 2016 included, 1234 LIFE funding events for environmental conservation were directed to 5,033 N2K sites, totalling 2.7 billion of Euros. The LIFE projects of the studied N2K sites targeted a large range of nature protection actions, mostly for wetland habitat and species conservation, and sometimes for other endangered habitats or species (e.g. lynx, raptors).

Designation period (n_Early_=1643, n_Late_=1375 IWC sites, range = 1982-2017). We used the first year reported among site classification, confirmation or designation, because some sites were already designated as PA under the Birds Directive before the N2K establishment in 1992. We compared early to late designation according to 2000, the mid-year of the 25-year monitoring period.

**Appendix 3. Sensitivity analyses.**

We conducted four additional sensitivity analyses (Appendix 3), to evaluate the robustness of our results to a number of analytical decisions. We checked: (1) whether CTI and CTI_SD_ trends were overly influenced by a few abundant species, by using species occurrence instead of abundance. (2) Whether the CTI and CTI_SD_ trends were affected by the geographical West-East EU accession gradient, by fitting models only with the subset of 11 countries in the EU before 1992 (n = 2,186 sites); (3) whether the community changes resulted from a decrease or an increase of species richness; and (4) whether the CTI trends associated with each N2K site protective action were correlated with the amount of protected wetland surface.

*Hypotheses and methods*

(1) The CTI and CTI_SD_ trends based on species abundance are usually similar to the trends based on species occurrence, but differences may suggest the impact of a few number of species (Devictor et al. 2008, Gaget et al. 2020b). The CTI and CTI_SD_ based on occurrence are the CTI and CTI_SD_ unweighted by species abundance (Gaget et al. 2020b).

(2) The temporal effect of the designation period before or after 2000 may be confounded with the geographical effect of the N2K North-East expansion due to EU country accession date. To investigate the potential influence of the geographical N2K extension we differentiated countries from their EU accession date. The monitored sites were located in 11 countries already inside the EU before 1992, Belgium, Denmark, France, Germany, Greece, Ireland, Italy, Netherlands, Portugal, Spain, and the United Kingdom (n = 2,186 sites).

We conducted the complementary analyses (1) and (2) by using linear mixed-effects models investigating the linear temporal change of CTI and CTI_SD_ (abundance and occurrence), using all the sites or only the sites within the 11 countries already in the UE before 1992. The models included the main effects of the N2K characteristics (waterbird target, management plan existence, LIFE funding and designation period), of the year, and the two-way interactions between N2K characteristics and year. The site and the country were added as a random effect and the spatial autocorrelation was taken into account by including the IWC site coordinates as an exponential spatial correlation structure in the model^25^.

(3) A CTI increase can be caused by an increasing abundance - and a colonization - of species with high STIs or a by a decreasing abundance - and an extinction - of species with low STIs.

To ensure that community changes were not driven by a decline of the species richness in an “extinction scenario”, we assessed in a generalised linear mixed model (Poisson error distribution) the temporal trend of the species richness in relation to the N2K site characteristics. Fixed effects were all main effects and the two-way interactions between N2K characteristics and year. The site and the country were added as random effects while the spatial autocorrelation was taken into account by including the IWC site coordinates with an exponential spatial correlation structure in the model^25^. The correlation between the CTI trend and the species richness trend over years per N2K characteristics (n = 16 combinations) was assessed with a Spearman correlation.

(4) We investigated the differences of N2K site wetland surfaces between site N2K characteristics (waterbird target, management plan existence, LIFE funding and designation period), assuming higher wetland surfaces inside N2K sites designated for protection of waterbirds. We also investigated whether higher wetland surfaces correlated with more positive CTI trends, assuming that higher wetland surfaces may increase both habitat diversity and availability, improving waterbird colonization notably by warm-dwelling species. The N2K site wetland surfaces were extracted from the N2K database (eunis.eea.europa.eu/sites). We considered wetlands as 18 different habitat classes: Inland water bodies (Standing water, Running water); Bogs, Marshes, Water fringed vegetation, Fens; Salt marshes, Salt pastures, Salt steppes; Tidal rivers, Estuaries, Mud flats, Sand flats, Lagoons (including saltwork basins); Marine areas, Sea inlets; Ricefields; Marine and coastal habitats (general). The surface per site was computed from the site size and the proportion of wetland habitats. The dataset used for wetland surfaces investigations include 2925 monitored sites (93 sites without habitat proportions were deleted from the data). Note that on average (±SD) 80.8 ± 23.3 % of the IWC surface was included in the corresponding N2K site (based on 1,307 IWC sites with available polygons).

We conducted the complementary analysis (4) by first investigating the differences of wetland surface relative to N2K characteristics levels using generalized linear models (negative binomial error distribution) with the wetland surface as response variable and the N2K characteristics as fixed effects. Second, we investigated the CTI (abundance) linear trend using the same CTI model as described in Methods, adding the main effect of the log(wetland surface) and its interaction with year.

*Results*

(1) The CTI and CTI_SD_ trends (Figure S1a and S1b) are fairly similar based on species occurrence (unfilled dots) or abundance (filled dots).

(2) The CTI and CTI_SD_ trends (Figure S1a and S1b)remain mostly unchanged considering all the EU countries (black dots) or only those already in the EU before 1992 (grey dots). The temperature trends were in general less positive in the EU Member States before 1992 (Figure S1b). However, the 95%CI of the CTI trends were considerably larger based on all countries (black dots) than on countries already in the EU before 1992 (grey dots) suggesting heterogeneous patterns of CTI trends in the countries that joint recently the EU, that could be due to geographical differences, including anthropogenic pressures (e.g. land-use change, harvesting) and differences of ecological characteristics (e.g. inland or coastal wetlands, species distribution).


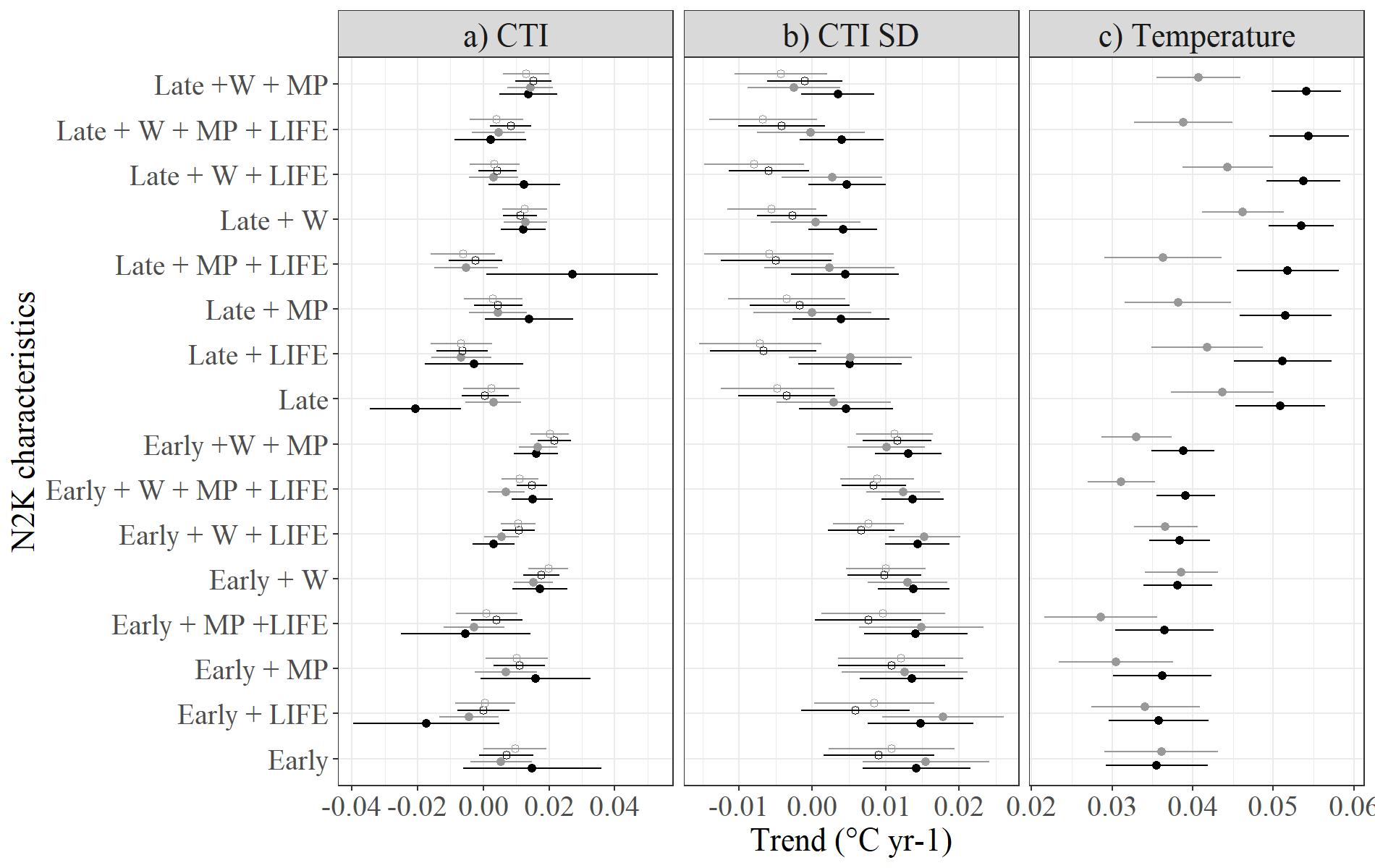


Figure S1: Parameter estimates (±95%CI) of the temporal trends of a) CTI, b) CTI_SD_ and c) temperature between N2K characteristics, based on all countries (black) or only the EU Member States before 1992 (grey), using abundance (filled dots) or occurrence data (unfilled dots). The N2K characteristics document whether waterbird(s) were targeted ([Yes/No]), a management plan has been prepared ([Yes/No]), LIFE funding has been obtained ([Yes/No]) and the period the protection was designated ([Early/Late], where early is <2000, the mid-year according to PA designation period (1982-2017). The EU Member States before 1992 were Belgium, Denmark, France, Germany, Greece, Ireland, Italy Netherlands, Portugal, Spain, and the United Kingdom.

(3) We find that in our case, a “colonisation scenario” is occurring, because the temporal trends of species richness per combination of N2K protective action were all positives (Figure S2) and correlated strongly to the CTI trends (Figure S3, r_Spearman_ = 0.92, p < 0.001).


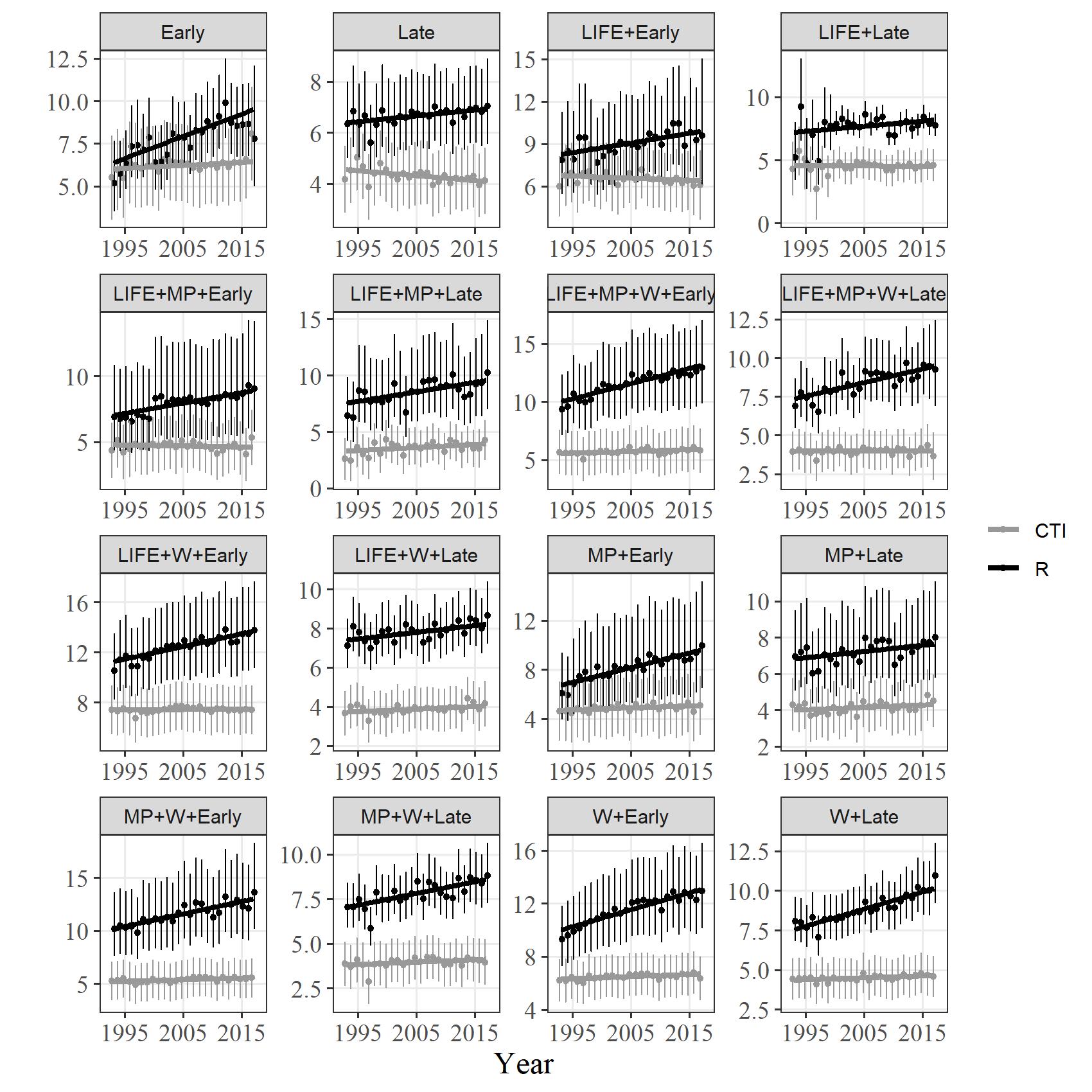


Figure S2: Temporal trend (±CI95%) of CTI (in grey) and species richness (R, in black) per combination of N2K protective action. Mean annual values (± 95% CI) have been generated by using the same model as for the linear trends, but changing year to a categorical variable.


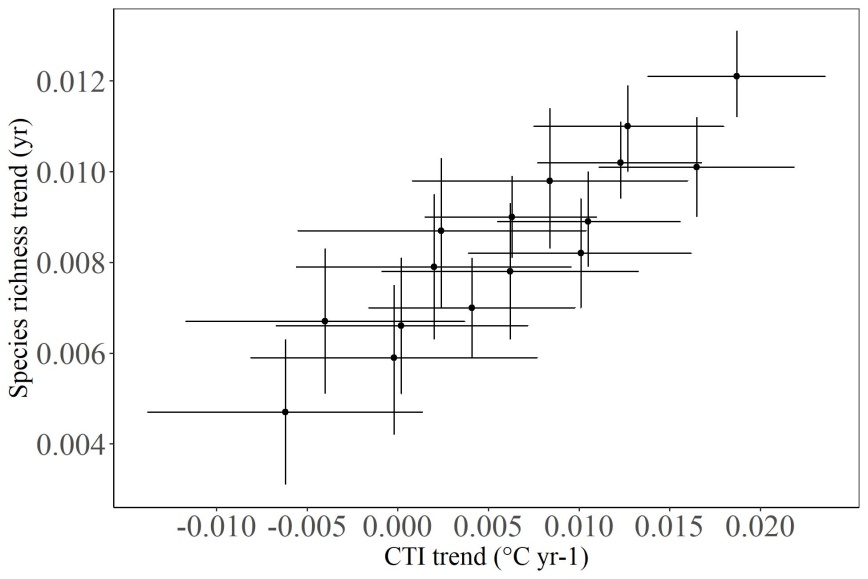


Figure S3. Temporal trend (±CI95%) of CTI and species richness per combination of N2K protective action. The temporal trends of species richness are all positives and correlated strongly to the CTI trends (r_Spearman_ = 0.92, p < 0.001).

(4) The wetland surface area was smaller in N2K sites designated for waterbird conservation (mean ± SD, 99 ± 414 km²) compared to sites without waterbird target (132 ± 870 km²) (p = 0.03); did not differ between sites with (105 ± 349 km²) or without management plan (106 ± 643 km²) (p = 0.5); was greater in sites with LIFE funding (104 ± 344 km²) than in sites without a LIFE funding (107 ± 644 km²) (p = 0.03); and was significantly smaller in sites designated before 2000 (78 ± 271 km²) than in sites designated since 2000 (140 ± 750 km²) (p < 0.001).

The wetland surfaces had a significant positive effect on the CTI average (F_1,2893_ = 123.1, p < 0.001) and temporal trend (F_1,34653_ = 9.0, p = 0.003). The results suggest that the community response to climate warming was higher when protected wetland surface next to the monitoring site was larger. The effects of the N2K characteristics on the CTI trend were similar than those estimated without adding the wetland surfaces in the model: CTI trends were more positive in protected sites targeted to protect waterbirds compared to protected sites not targeted to protect waterbirds (F_1,34653_ = 8.4, p = 0.004); more positive in protected sites with a management plan compared to CTI change in protected sites without a management plan (F_1,34653_ = 4.9, p = 0.03). Furthermore, CTI trends were more positive in protected sites without LIFE funding compared to CTI change in protected sites with LIFE funding (F_1,34653_ = 7.2, p = 0.002). Last, the CTI change in N2K sites established prior to 2000 (“early”) did not differ from N2K sites established after 2000 (F_1,34653_ < 0.0, p = 0.9).

Appendix 4. Pairwise comparisons of the CTI temporal trends according to the N2K characteristics; waterbird(s) were targeted (W), a management plan has been prepared (MP), the period the protection was designated (Late or Early, where early is <2000), or LIFE funding has been obtained (LIFE). The significant differences, after Bonferroni correction, are denoted in bold (α<0.05).

| Pairwaise comparisons | | | Estimate | SE | df | t | p |
| --- | --- | --- | --- | --- | --- | --- | --- |
| Early | / | Early + MP | -0.006 | 0.003 | 35536 | -2.355 | 1.000 |
| Early | / | Early + W | -0.010 | 0.003 | 35536 | -2.983 | 0.343 |
| **Early** | **/** | **Early + W + MP** | **-0.016** | **0.004** | **35536** | **-3.777** | **0.019** |
| Early | / | Late | 0.002 | 0.003 | 35536 | 0.812 | 1.000 |
| Early | / | Late + MP | -0.004 | 0.004 | 35536 | -0.959 | 1.000 |
| Early | / | Late + W | -0.008 | 0.005 | 35536 | -1.711 | 1.000 |
| Early | / | Late + W + MP | -0.014 | 0.006 | 35536 | -2.529 | 1.000 |
| Early + LIFE | / | Early | -0.006 | 0.003 | 35536 | -2.483 | 1.000 |
| Early + LIFE | / | Early + MP | -0.012 | 0.004 | 35536 | -3.495 | 0.057 |
| Early + LIFE | / | Early + MP + LIFE | -0.006 | 0.003 | 35536 | -2.355 | 1.000 |
| **Early + LIFE** | **/** | **Early + W** | **-0.017** | **0.004** | **35536** | **-3.815** | **0.016** |
| Early + LIFE | / | Early + W + LIFE | -0.010 | 0.003 | 35536 | -2.983 | 0.343 |
| **Early + LIFE** | **/** | **Early + W + MP** | **-0.023** | **0.005** | **35536** | **-4.511** | **0.001** |
| **Early + LIFE** | **/** | **Early + W + MP + LIFE** | **-0.016** | **0.004** | **35536** | **-3.777** | **0.019** |
| Early + LIFE | / | Late | -0.004 | 0.003 | 35536 | -1.290 | 1.000 |
| Early + LIFE | / | Late + LIFE | 0.002 | 0.003 | 35536 | 0.812 | 1.000 |
| Early + LIFE | / | Late + MP | -0.010 | 0.004 | 35536 | -2.367 | 1.000 |
| Early + LIFE | / | Late + MP + LIFE | -0.004 | 0.004 | 35536 | -0.959 | 1.000 |
| Early + LIFE | / | Late + W | -0.015 | 0.005 | 35536 | -2.819 | 0.579 |
| Early + LIFE | / | Late + W + LIFE | -0.008 | 0.005 | 35536 | -1.711 | 1.000 |
| Early + LIFE | / | Late + W + MP | -0.021 | 0.006 | 35536 | -3.486 | 0.059 |
| Early + LIFE | / | Late + W + MP + LIFE | -0.014 | 0.006 | 35536 | -2.529 | 1.000 |
| Early + MP | / | Early + W + MP | -0.010 | 0.003 | 35536 | -2.983 | 0.343 |
| Early + MP | / | Late + MP | 0.002 | 0.003 | 35536 | 0.812 | 1.000 |
| Early + MP | / | Late + W + MP | -0.008 | 0.005 | 35536 | -1.711 | 1.000 |
| Early + MP + LIFE | / | Early | 0.000 | 0.004 | 35536 | -0.122 | 1.000 |
| Early + MP + LIFE | / | Early + MP | -0.006 | 0.003 | 35536 | -2.483 | 1.000 |
| Early + MP + LIFE | / | Early + W | -0.011 | 0.005 | 35536 | -2.106 | 1.000 |
| **Early + MP + LIFE** | **/** | **Early + W + MP** | **-0.017** | **0.004** | **35536** | **-3.815** | **0.016** |
| Early + MP + LIFE | / | Early + W + MP + LIFE | -0.010 | 0.003 | 35536 | -2.983 | 0.343 |
| Early + MP + LIFE | / | Late | 0.002 | 0.004 | 35536 | 0.436 | 1.000 |
| Early + MP + LIFE | / | Late + MP | -0.004 | 0.003 | 35536 | -1.290 | 1.000 |
| Early + MP + LIFE | / | Late + MP + LIFE | 0.002 | 0.003 | 35536 | 0.812 | 1.000 |
| Early + MP + LIFE | / | Late + W | -0.009 | 0.006 | 35536 | -1.525 | 1.000 |
| Early + MP + LIFE | / | Late + W + MP | -0.015 | 0.005 | 35536 | -2.819 | 0.579 |
| Early + MP + LIFE | / | Late + W + MP + LIFE | -0.008 | 0.005 | 35536 | -1.711 | 1.000 |
| Early + W | / | Early + MP | 0.004 | 0.004 | 35536 | 1.012 | 1.000 |
| Early + W | / | Early + W + MP | -0.006 | 0.003 | 35536 | -2.355 | 1.000 |
| Early + W | / | Late + MP | 0.006 | 0.005 | 35536 | 1.324 | 1.000 |
| Early + W | / | Late + W | 0.002 | 0.003 | 35536 | 0.812 | 1.000 |
| Early + W | / | Late + W + MP | -0.004 | 0.004 | 35536 | -0.959 | 1.000 |
| Early + W + LIFE | / | Early | 0.004 | 0.004 | 35536 | 0.909 | 1.000 |
| Early + W + LIFE | / | Early + MP | -0.002 | 0.005 | 35536 | -0.436 | 1.000 |
| Early + W + LIFE | / | Early + MP + LIFE | 0.004 | 0.004 | 35536 | 1.012 | 1.000 |
| Early + W + LIFE | / | Early + W | -0.006 | 0.003 | 35536 | -2.483 | 1.000 |
| Early + W + LIFE | / | Early + W + MP | -0.012 | 0.004 | 35536 | -3.495 | 0.057 |
| Early + W + LIFE | / | Early + W + MP + LIFE | -0.006 | 0.003 | 35536 | -2.355 | 1.000 |
| Early + W + LIFE | / | Late | 0.006 | 0.004 | 35536 | 1.392 | 1.000 |
| Early + W + LIFE | / | Late + MP | 0.000 | 0.005 | 35536 | 0.013 | 1.000 |
| Early + W + LIFE | / | Late + MP + LIFE | 0.006 | 0.005 | 35536 | 1.324 | 1.000 |
| Early + W + LIFE | / | Late + W | -0.004 | 0.003 | 35536 | -1.290 | 1.000 |
| Early + W + LIFE | / | Late + W + LIFE | 0.002 | 0.003 | 35536 | 0.812 | 1.000 |
| Early + W + LIFE | / | Late + W + MP | -0.010 | 0.004 | 35536 | -2.367 | 1.000 |
| Early + W + LIFE | / | Late + W + MP + LIFE | -0.004 | 0.004 | 35536 | -0.959 | 1.000 |
| Early + W + MP | / | Late + W + MP | 0.002 | 0.003 | 35536 | 0.812 | 1.000 |
| Early + W + MP + LIFE | / | Early | 0.010 | 0.005 | 35536 | 1.960 | 1.000 |
| Early + W + MP + LIFE | / | Early + MP | 0.004 | 0.004 | 35536 | 0.909 | 1.000 |
| Early + W + MP + LIFE | / | Early + W | 0.000 | 0.004 | 35536 | -0.122 | 1.000 |
| Early + W + MP + LIFE | / | Early + W + MP | -0.006 | 0.003 | 35536 | -2.483 | 1.000 |
| Early + W + MP + LIFE | / | Late | 0.012 | 0.005 | 35536 | 2.448 | 1.000 |
| Early + W + MP + LIFE | / | Late + MP | 0.006 | 0.004 | 35536 | 1.392 | 1.000 |
| Early + W + MP + LIFE | / | Late + W | 0.002 | 0.004 | 35536 | 0.436 | 1.000 |
| Early + W + MP + LIFE | / | Late + W + MP | -0.004 | 0.003 | 35536 | -1.290 | 1.000 |
| Early + W + MP + LIFE | / | Late + W + MP + LIFE | 0.002 | 0.003 | 35536 | 0.812 | 1.000 |
| Late | / | Early + MP | -0.008 | 0.003 | 35536 | -2.380 | 1.000 |
| Late | / | Early + W | -0.012 | 0.004 | 35536 | -3.128 | 0.211 |
| **Late** | **/** | **Early + W + MP** | **-0.018** | **0.005** | **35536** | **-4.063** | **0.006** |
| Late | / | Late + MP | -0.006 | 0.003 | 35536 | -2.355 | 1.000 |
| Late | / | Late + W | -0.010 | 0.003 | 35536 | -2.983 | 0.343 |
| **Late** | **/** | **Late + W + MP** | **-0.016** | **0.004** | **35536** | **-3.777** | **0.019** |
| Late + LIFE | / | Early | -0.009 | 0.004 | 35536 | -2.083 | 1.000 |
| Late + LIFE | / | Early + MP | -0.015 | 0.005 | 35536 | -3.179 | 0.177 |
| Late + LIFE | / | Early + MP + LIFE | -0.008 | 0.003 | 35536 | -2.380 | 1.000 |
| **Late + LIFE** | **/** | **Early + W** | **-0.019** | **0.005** | **35536** | **-3.682** | **0.028** |
| Late + LIFE | / | Early + W + LIFE | -0.012 | 0.004 | 35536 | -3.128 | 0.211 |
| **Late + LIFE** | **/** | **Early + W + MP** | **-0.025** | **0.006** | **35536** | **-4.503** | **0.001** |
| **Late + LIFE** | **/** | **Early + W + MP + LIFE** | **-0.018** | **0.005** | **35536** | **-4.063** | **0.006** |
| Late + LIFE | / | Late | -0.006 | 0.003 | 35536 | -2.483 | 1.000 |
| Late + LIFE | / | Late + MP | -0.012 | 0.004 | 35536 | -3.495 | 0.057 |
| Late + LIFE | / | Late + MP + LIFE | -0.006 | 0.003 | 35536 | -2.355 | 1.000 |
| **Late + LIFE** | **/** | **Late + W** | **-0.017** | **0.004** | **35536** | **-3.815** | **0.016** |
| Late + LIFE | / | Late + W + LIFE | -0.010 | 0.003 | 35536 | -2.983 | 0.343 |
| **Late + LIFE** | **/** | **Late + W + MP** | **-0.023** | **0.005** | **35536** | **-4.511** | **0.001** |
| **Late + LIFE** | **/** | **Late + W + MP + LIFE** | **-0.016** | **0.004** | **35536** | **-3.777** | **0.019** |
| Late + MP | / | Early + W + MP | -0.012 | 0.004 | 35536 | -3.128 | 0.211 |
| Late + MP | / | Late + W + MP | -0.010 | 0.003 | 35536 | -2.983 | 0.343 |
| Late + MP + LIFE | / | Early | -0.003 | 0.005 | 35536 | -0.517 | 1.000 |
| Late + MP + LIFE | / | Early + MP | -0.009 | 0.004 | 35536 | -2.083 | 1.000 |
| Late + MP + LIFE | / | Early + W | -0.013 | 0.006 | 35536 | -2.183 | 1.000 |
| **Late + MP + LIFE** | **/** | **Early + W + MP** | **-0.019** | **0.005** | **35536** | **-3.682** | **0.028** |
| Late + MP + LIFE | / | Early + W + MP + LIFE | -0.012 | 0.004 | 35536 | -3.128 | 0.211 |
| Late + MP + LIFE | / | Late | 0.000 | 0.004 | 35536 | -0.122 | 1.000 |
| Late + MP + LIFE | / | Late + MP | -0.006 | 0.003 | 35536 | -2.483 | 1.000 |
| Late + MP + LIFE | / | Late + W | -0.011 | 0.005 | 35536 | -2.106 | 1.000 |
| **Late + MP + LIFE** | **/** | **Late + W + MP** | **-0.017** | **0.004** | **35536** | **-3.815** | **0.016** |
| Late + MP + LIFE | / | Late + W + MP + LIFE | -0.010 | 0.003 | 35536 | -2.983 | 0.343 |
| Late + W | / | Early + MP | 0.002 | 0.005 | 35536 | 0.410 | 1.000 |
| Late + W | / | Early + W + MP | -0.008 | 0.003 | 35536 | -2.380 | 1.000 |
| Late + W | / | Late + MP | 0.004 | 0.004 | 35536 | 1.012 | 1.000 |
| Late + W | / | Late + W + MP | -0.006 | 0.003 | 35536 | -2.355 | 1.000 |
| Late + W + LIFE | / | Early | 0.002 | 0.006 | 35536 | 0.296 | 1.000 |
| Late + W + LIFE | / | Early + MP | -0.004 | 0.006 | 35536 | -0.725 | 1.000 |
| Late + W + LIFE | / | Early + MP + LIFE | 0.002 | 0.005 | 35536 | 0.410 | 1.000 |
| Late + W + LIFE | / | Early + W | -0.009 | 0.004 | 35536 | -2.083 | 1.000 |
| Late + W + LIFE | / | Early + W + MP | -0.015 | 0.005 | 35536 | -3.179 | 0.177 |
| Late + W + LIFE | / | Early + W + MP + LIFE | -0.008 | 0.003 | 35536 | -2.380 | 1.000 |
| Late + W + LIFE | / | Late | 0.004 | 0.004 | 35536 | 0.909 | 1.000 |
| Late + W + LIFE | / | Late + MP | -0.002 | 0.005 | 35536 | -0.436 | 1.000 |
| Late + W + LIFE | / | Late + MP + LIFE | 0.004 | 0.004 | 35536 | 1.012 | 1.000 |
| Late + W + LIFE | / | Late + W | -0.006 | 0.003 | 35536 | -2.483 | 1.000 |
| Late + W + LIFE | / | Late + W + MP | -0.012 | 0.004 | 35536 | -3.495 | 0.057 |
| Late + W + LIFE | / | Late + W + MP + LIFE | -0.006 | 0.003 | 35536 | -2.355 | 1.000 |
| Late + W + MP + LIFE | / | Early | 0.008 | 0.006 | 35536 | 1.198 | 1.000 |
| Late + W + MP + LIFE | / | Early + MP | 0.002 | 0.006 | 35536 | 0.296 | 1.000 |
| Late + W + MP + LIFE | / | Early + W | -0.003 | 0.005 | 35536 | -0.517 | 1.000 |
| Late + W + MP + LIFE | / | Early + W + MP | -0.009 | 0.004 | 35536 | -2.083 | 1.000 |
| Late + W + MP + LIFE | / | Late | 0.010 | 0.005 | 35536 | 1.960 | 1.000 |
| Late + W + MP + LIFE | / | Late + MP | 0.004 | 0.004 | 35536 | 0.909 | 1.000 |
| Late + W + MP + LIFE | / | Late + W | 0.000 | 0.004 | 35536 | -0.122 | 1.000 |
| Late + W + MP + LIFE | / | Late + W + MP | -0.006 | 0.003 | 35536 | -2.483 | 1.000 |
